# Exclusion Zone Phenomena in Water—A Critical Review of Experimental Findings and Theories

**DOI:** 10.3390/ijms21145041

**Published:** 2020-07-17

**Authors:** Daniel C. Elton, Peter D. Spencer, James D. Riches, Elizabeth D. Williams

**Affiliations:** 1 Radiology and Imaging Sciences, National Institutes of Health Clinical Center, Bethesda, MD 20892, USA; 2 School of Biomedical Sciences, Faculty of Health, Institute of Health and Biomedical Innovation, Queensland University of Technology (QUT), Brisbane, QLD 4059, Australia; 3 School of Earth, Environmental and Biological Sciences, Science and Engineering Faculty, Institute for Future Environments, QUT, Brisbane, QLD 4000, Australia; jamie.riches@qut.edu.au; 4 School of Biomedical Sciences, Faculty of Health, Institute of Health and Biomedical Innovation, QUT, Australian Prostate Cancer Research Centre—Queensland, Translational Research Institute, Brisbane, QLD 4059, Australia; ed.williams@qut.edu.au

**Keywords:** water, exclusion zone, diffusiophoresis, repulsive van der Waals

## Abstract

The existence of the exclusion zone (EZ), a layer of water in which plastic microspheres are repelled from hydrophilic surfaces, has now been independently demonstrated by several groups. A better understanding of the mechanisms which generate EZs would help with understanding the possible importance of EZs in biology and in engineering applications such as filtration and microfluidics. Here we review the experimental evidence for EZ phenomena in water and the major theories that have been proposed. We review experimental results from birefringence, neutron radiography, nuclear magnetic resonance, and other studies. Pollack theorizes that water in the EZ exists has a different structure than bulk water, and that this accounts for the EZ. We present several alternative explanations for EZs and argue that Schurr’s theory based on diffusiophoresis presents a compelling alternative explanation for the core EZ phenomenon. Among other things, Schurr’s theory makes predictions about the growth of the EZ with time which have been confirmed by Florea et al. and others. We also touch on several possible confounding factors that make experimentation on EZs difficult, such as charged surface groups, dissolved solutes, and adsorbed nanobubbles.

## 1. Introduction

Prof. Gerald Pollack’s group has provided many convincing experimental demonstrations of an exclusion zone (EZ) in water whereby particles such as plastic microspheres are repelled from a surface. The width of the EZ depends on the properties of the surface and ambient conditions and may reach hundreds of microns. In addition to small particles, there is evidence that the EZ excludes relatively large molecules such as pH-indicators and biological molecules.

For the case of highly hydrophillic surfaces these findings have now been reproduced by several independent research groups [1,2,3,4,5,6,7,8,9,10,11] and constitute a genuine physical phenomena which is in need of a theoretical explanation. A few experimenters have reported EZs near metal surfaces [12,13,14], and one has reported it for cellulose [15]. In this work we present a review of exclusion zone phenomena, including many recent experimental studies, and describe several mechanisms by which the EZ phenomena can arise. In any given experimental scenario, some or all of those mechanism may be present. EZ phenomena may have important engineering applications in water filtration, reducing biofouling [16], and microfluidics [6]. EZ phenomena also have obvious importance to understanding biological systems and resolving outstanding questions about “biological water” [17].

## 2. Background

The existence of structured water near hydrophilic interfaces has been proposed several times previously. Drost-Hansen (1969, 1973) reviewed many experiments and came to the conclusion that interfacial (“vicinal”) water exhibits structural difference that extend to tens to thousands of molecular diameters [18,19]. A common theme found in the literature is that hydrophillic surfaces result in a change in the structure of interfacial water due to “templating” of the surface water molecules [20,21,22]. Many claims for ordering near biological interfaces (i.e., in cells or small blood vessels) have been made, with many positing that “biological water” has significant structural differences [23]. One of the earliest studies in this vein was performed by Deryagin in 1986, who also described an EZ type phenomena in cells [6,24]. A difficulty in such research is separating out property changes that occur due to confinement, which are largely thermodynamic in nature (i.e., from Laplace pressure), from effects due to the putative restructuring of cellular water. Despite many works on “biological water”, the hypothesis that cellular water undergoes significant restructuring remains very controversial (for a review, see Ball, 2008) [17]. It is not our intent to review that controversy here, but only to highlight its relationship to the EZ water controversy.

At a hydrophilic surface, the alignment of hydrogen bonds at the surface may create a polarized layer and electric field, the influence of which may extend out for several layers of water molecules. This argument has been used to support both experimental evidence from X-ray and spectroscopic studies for order at the water-hydrophilic surface interface [20,25,26,27]. While this ordering is often called “long-range”, the extend found in most studies is only a few water layers (i.e., 1–2 nm). This level of restructuring, which extends just a few molecular layers, is consistent with the predictions of double layer theory [20] and molecular dynamics studies quantifying the extent of angular correlation in the bulk and near interfaces [28,29,30]. The limited extent of restructuring is not surprising given that hydrogen bonds are relatively weak (0.24 eV per bond) and are short lived due to thermal perturbations (lifetime ≈ 1 ps) [27,31].

## 3. Pollack’s Key Experimental Findings and Replications

The exclusion zone was first described by Pollack et al. in 2003 after they observed latex microspheres in suspension moving away from the surface of the hydrophilic material Nafion (a sulfonated tetrafluoroethylene based fluoropolymercopolymer developed by DuPont) under a microscope [32]. Using UV-vis absorption spectra and NMR, in 2006 Pollack et al. argued that EZ water exists in a different phase [12]. Further investigations from Pollack’s lab in 2007 using microelectrodes indicated that the EZ region is negatively charged [33]. This is consistent with Electrical Impedance Spectroscopy studies of Nafion, which measure the electrical potential of water near Nafion by passing an alternating current of known frequency and small amplitude through it [34]. On the other hand, experiments by Chai, Mahtani, and Pollack (2012) showed that EZs near the charged surfaces of some metals are positively charged [14]. On the other hand, experiments by Chai, Mahtani, and Pollack (2012) showed that EZs near the charged surfaces of some metals are positively charged [14]. Additionally, water in the EZ was reported to have a higher index of refraction, which is attributed to a higher density due to a change in the water’s structure [35]. Hwang et al. attempted to measure the increase in density by dissolving a hydrophilic ceramic powder in water and then filtering the water, but only a small (0.4%) increase was observed [36]. While most experiments have been done showing exclusion of microspheres, one experiment from Pollack’s lab shows rejection of salt as well [37]. Introduction of pH sensitive dye indicated a low pH (<3) close to the Nafion surface, as well as a small region very close to the surface where the dye appeared to be excluded [38]. A summary of properties that have been reported for EZ water can be found in Table 1.

## 4. The Structure Change Theory

A few researchers have proposed that the EZ is due to a change in water’s structure [12,39,40,41,42,43,44]. One problem with this is there are no obvious thermodynamic forces in the system to drive such a phase change or long range ordering, and such effects are not observed in conventional molecular dynamics simulations. Giudice et al. propose that quantum electrodynamics calculations are necessary to understand the phase transition which occurs in EZ water [42,44,45], a claim that is forcefully disputed in a detailed work by Bier and Pravaca [46]. In his popular science book “The Fourth Phase of Water”, Pollack hypothesizes that the EZ water is structured in hexagonal sheets, with the hydrogens lying directly between oxygens [35]. Pollack proposes that when these sheets are stacked hydrogen atoms bond to the oxygens in neighboring layers, such that each hydrogen forms three bonds. It is important to note that his book is not peer reviewed and not a scientific monograph, and Pollack admits that the idea of a layered structure is speculative [35]. In other work, Pollack has proposed that the structure is an intermediate between ice and water [47]. Oehr and LeMay (2014) present a similar theory that the observed EZ water may comprise tetrahedral oxy-subhydride structures [41]. It is worth noting that in 1962 Fedayakin proposed that “polywater” (a type of water purported to exist in capillary tubes) had a similar honeycomb like structure with each oxygen bonded to 3 hydrogens, similar to both Pollack’s and Oehr and LeMay’s structures [48]. In response to this, in 1971 Hasted noted problems with hexagonal water structures in general, noting that high energy cost of placing hydrogens between oxygens was enough to make such structures explode if they were ever created [49]. Much more recently Seggara-Martí et al. performed quantum chemistry calculations showing such a structure to be unstable [50]. Further quantum chemistry calculations were performed on two stacked hexagonal layers (each layer contained two hexagons and one negative change (H${}_{19}$O${}_{10}^{-}$). The negative charge did not distribute uniformly over the structure and optimization of the structure resulted in a “bulk-type water aggregate”, showing it to be unstable [50].

Elia et al. suggest that perturbations near the EZ surface can cause clumps of EZ water to disperse in the bulk liquid, resulting in changes that can be detected in the bulk liquid as the EZ water clumps dissipate [43]. Figuera and Pollack have presented a somewhat similar argument, arguing that the stable nature of the EZs under perturbations must be due to a structure change [40].

Exclusion zone phenomena have been observed in other polar liquids as well such as dimethyl sulfoxide (DMSO), suggesting that hydrogen bonds are not required for the phenomena [51]. If it were the case that EZs were due to a phase change we would expect EZ phenomena would be quite different between water, which supports low density hexagonal structures and hydrogen bonding, and other polar solvents which do not. As we discuss in the next section, neutron radiography does not support the notion of a higher density phase. An experiment which could shed additional light on this subject is X-ray crystalography. X-ray crystalography has not been done for the EZ but has been used to examine the electrically-induced water bridge which Pollack hypothesizes may be made of EZ water in his popular science book *The Fourth Phase of Water: Beyond Solid, Liquid, and Vapor* (2013). Both molecular dynamics simulation [52], X-ray crystallography [52], and neutron scattering [53] show that the internal structure of the water bridge is unchanged—implying that it is supported by enhanced surface tension rather than a change in internal structure.

Pollack points to enhanced absorption at 270 nm as evidence for a possible phase change in the EZ [12,47]. This absorption peak was not found in quantum chemistry simulations [50]. Strikingly, results from Pollack’s own lab show that a similar absorption peak is seen in pure salt solutions (LiCl, NaCl, KCl), so the source of this enhanced absorption appears to be related to dissolved solutes [54]. A study of Arrowhead Spring found absorption at 270 nm in bulk water [55]. Hypothesizing that EZ water would be a transitionary form between ice and liquid water, Pollack performed IR measurements of melting ice [47]. During the course of these experiments the 270 nm peak sometimes (but not always) appeared transiently (i.e., for a few seconds) while the ice was melting. In the same work they also report that degassing the water (either through boiling, drawing a vacuum, or nitrogen bubbling) reduced of the appearance of the peak [47]. Thus, it is also possible that the peak is related to tiny bubbles trapped in the ice which migrate to the surface while the ice is melting. A possible mechanism which why nanobubbles are responsible for absorption near 270 nm would absorption from superoxide anions (O${}^{-2}$) and their protonated form, the hydroperoxyl radical (HO${}_{2}$). These two species exist in equilibrium in small quantities when oxygen is disolved in water, exist in much higher quantifies in acidic solutions, and can be induced by UV radiation [56]. There is evidence that both species absorb in the range of 240–260 nm [56,57,58,59].

Pollack also hypothesizes that when light is shined on EZ water it causes positive and negative charges to separate, and the EZ water region to grow [38]. This is problematic since water is a good conductor and charge separation would be difficult to sustain. Base on this idea, speculative hypotheses that EZ water is important for cellular energy production and biological function have been explored by a number of researchers [10,60,61,62].

### 4.1. Testing the Structure Change Theory with Neutron Radiography

**Figure 1:**
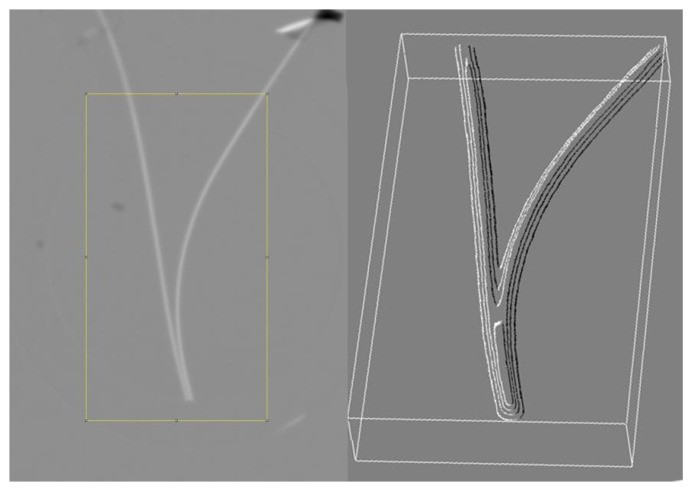
Image produced by subtracting the natural logarithm of the neutron attenuation in the distilled water filled cell with and without two strips of Nafion. The yellow outline shows the region of interest for creating the 3D surface plot shown on the right.

As described in detail in Reference [63], some of the authors on this work recently undertook a neutron radiography study to measure the density of water near the Nafion surface. Pollack’s proposed EZ water structure has a density which is ≈ 10% higher than liquid water. Neutron radiography has previously been used to measure subtle density differences between supercritical and subcritical water [64]. The experiment was conducted using the Dingo radiography imaging station at the Australian Nuclear Science and Technology Organization (ANSTO). The neutron flux varied between 1.14 × 10${}^{7}$ to 4.75 × 10${}^{7}$ neutrons cm${}^{2}$ s${}^{-1}$. Imaging with test objects indicated the instrumental resolution was at least 100 μm, which is adequate to detect an EZ extent of 200 μm, smaller than the extent of 500+ μm proposed by Pollack and collaborators [12,65]. In the experiment, a 2 mm wide quartz glass cell was filled with distilled water and two strips of Nafion were inserted. The temperature was held at 21 ${}^{{}^{\circ}}$C ± 1 ${}^{{}^{\circ}}$C. and the Nafion strips were 0.43 mm thick and 1–2 mm in width. It was expected that a denser region of EZ water would nucleate from the Nafion surface, resulting in greater neutron attenuation. The arrangement of the two Nafion strips in a “V” formation was intended to create an effect where the visible difference due to EZ formation could be doubled, creating an EZ region large enough to be identified between the strips. Figure 1 shows the difference between the natural logarithm of attenuation in the cell with and without two strips of Nafion. As can be clearly seen, no density differences are observable near the surface, at least within the 100 μm resolution of the instrument. A nuclear magnetic resonance (NMR) study has showed water polarization and ordering next to fused silica (an allotrope of quartz), but the extent of this ordering was found to be limited to 60 molecular layers [66]. Thus it can be concluded that EZs do not form near quartz to begin with [66].

### 4.2. Testing for Structure Change with Optical Birefringence Measurement

Another piece of experimental evidence that Pollack presents for EZ water having a different structure is the presence of optical birefringence in the EZ caused by Nafion [35,67]. Attempts to replicate this result was performed by some of the authors using a polarized light microscope setup [9,63]. It was found that there are confounding factors which cause the appearance of birefringence near the surface of Nafion. Both air-dried Nafion and zinc still exhibited a high degree of birefringence near the surface due to light reflected obliquely from the surface [9]. The way that the surface was cut also changed the degree of reflection birefringence observed, with a blade cut surface showing more of this effect than a rough surface cut with scissors. In addition, in some cases microspheres reflect light and thus give the appearance of a wide birefringent region extending from the material surface into the bulk water [63]. In a similar vein, polarization by reflection has been noted to play a confounding role in the measurement of the birefringence properties of ice [68]. Thus, the measurements of birefringence near the surfaces of Nafion, zinc, and other metals were due to optical effects from uncontrolled-for reflections and do not constitute an evidence for underlying crystalline ordering in water. Another experiment which may suffer from similar confounds is the work of Bunkin et al. and Tychinsky which measured an increased refractive index of water very close to the surface of Nafion [7,69].

## 5. Alternative Explanations for EZ Phenomena

This section presents several alternative explanations to EZ phenomena—diffusiophoresis (long range chemotaxis), reported previously by Schurr, and van der Waals forces. These theories provide quantitative explanations for the growth and maintenance of the exclusion zone where plastic microspheres made of (possibly functionalized) carboxylate, polystyrene, amidine, or polytetrafluoroethylene (PTFE) are repelled from various surfaces.

### 5.1. Diffusiophoresis

**Figure 2:**
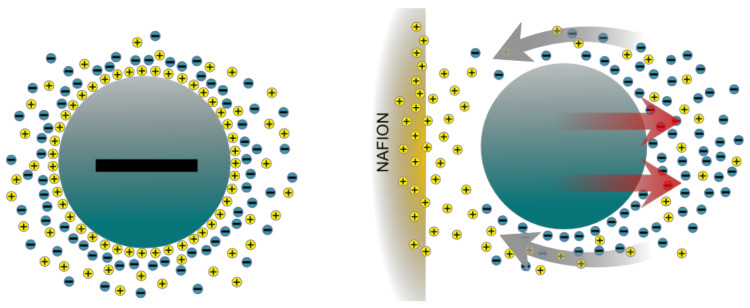
(**left**) Homogeneous case. (**right**) Heterogeneous case leading to diffusiophoresis.

Schurr (2013) has developed a theory which proposes that the EZ formation is created by forces arising from a concentration gradients of OH${}^{-}$ or H${}^{+}$ and salt. Called “long range chemotaxis” by Schurr [70,71], it is a type of a more general and well known phenomena in colloid science called diffusiophoresis. Huyghe, Wyss et al. (2014) propose that the EZs are generated by a combination of ion exchange and diffusiophoresis [2]. They note that Nafion has an ample supply of exchangeable protons ready to exchange with cations in the solution. Such an exchange would create an inhomogeneous distribution of ions (salt gradient) in the liquid. According to the diffusiophoresis theory, a charged particle in an electrolyte solution would attract counter-ions (oppositely charged) via the influence of the local electric field. In a homogeneous solution it would be expected that the distribution of ions and counter-ions would be symmetrical around the particle. This would lead to a homogeneously distributed hydrostatic pressure with no fluid flow as shown in the left side of Figure 2. However, with the introduction of a proton donor like Nafion the resulting inhomogeneous charge distribution would produce an asymmetrical arrangement of ions around the particle as shown in the right side of Figure 2. In an effort to balance ions and counter ions a fluid flow results, propelling the particles away from the Nafion surface.

Florea et al. have performed experiments on the EZ, carefully measuring its time course, and have shown that the data are fit by their model of diffusiophoresis [6]. Notably, these experiments were done with the hydrophilic surface horizontal, which avoids convective fluid motions due to the force of gravity which occur when it is vertical, as in many of Pollack’s experiments. Further experiments and a computational study using COMSOL Multiphysics simulation by Esplandiu et al. lend further support to the findings of Florea et al. [11]. Huszár et al. note that the growth of the exclusion zone with times follows a power law with an exponent of 0.6, very close to the exponent of 0.5 expected for a diffusion-driven process [3]. Using laser tweezers, a forcefield has been measured inside the exclusion zone. Two independent experiments have found that the magnitude of the repulsive force decays as a function of distance from the surface in a manner consistent with the diffusiophoresis theory [1,3]. The presence of a force decaying from the surface is inconsistent with the theory that a new phase forms in the exclusion zone.

Pollack has responded to Shurr’s original work [72]. Figure 1 in Pollack’s response arguably support the theory however, since it shows a large pH gradient, as indicated by a dye [72]. However, in an earlier work Ovchinnikova & Pollack argue that the pH gradients reflect storage and slow dissipation of electric charge by the EZ water rather than the Nafion [73].

Apart from the experiments mentioned previously, there are theoretical reasons to suppose that a large concentration gradient would arise near the surface of Nafion, the most popular surface used for generating EZs. Nafion is a copolymer of tetrafluoroethylene and perfluoro- 3,6-dioxa-4-methyl-7-octene sulfonic acid which finds application in fuel cell technology. If the sulfonic acid part were allowed to dissolve into water it would be quite a strong acid, but this does not happen since it remains bonded into the copolymer. When Nafion is placed in water it quickly swells, resulting in a gel-structure with an extremely high surface area. In this structure all of the sulphonic acid groups are surrounded by water. The highly negative sufonic acid group dissociates water and adsorbs H+ ions, resulting in a very low internal pH for Nafion, as observed with indicators such as methylene blue [74]. Computational studies show it is energetically favorable for 2–4 hydronium ions to surround each sulfonic acid group [75,76]. Using methylene blue the internal acidity of Nafion has been estimated to be equivalent to 1.2 M sulphuric acid [74]. The excess protons inside Nafion are of two types—“fixed” ions which can “hop” between sulfonic groups, and “mobile” ions which can can freely diffuse away [74,76]. Thus water around Nafion becomes acidic, with a pH gradient approaching neutral (7) further away from the memberane. This is shown clearly in experiments by Pollack where pH sensitive dyes have been added to the water [38]. We have also observed this in our own experiments, were we also found that the average pH of the water around Nafion drops over the course of several days [9,63].Elsewhere an acidic pH of water around Nafion has also been reported (pK${}_{a}$ ≈ −6) [77].

### 5.2. EZs at Metal Surfaces: Van Der Waals Repulsion and Quantum Phenomena

The theory of chemotaxis of Schurr presents a compelling theory of the EZ phenomena observed near Nafion. However, Pollack’s group has also reported EZ phenomena near metal surfaces, although they are much smaller in size [14]. The EZ is largest for Zinc (220 μm), followed by aluminum, lead, tin, and tungsten (72 μm) [14]. Notably however, attempts to independently replicate these findings with aluminum and zinc have failed [9]. Pollack also reports EZ phenomena at the surface of platinum, but only after a voltage is applied [78]. While water molecules adsorb onto surfaces like platinum [13], and may dissociate on such surfaces in certain circumstances [79], the expected gradient of hydronium ions as one moves away from the surface is expected to be small, if it exists at all. One possibility is that the exclusion zone phenomena near metals (and possibly other materials) may be partially explained by repulsive van der Waals forces (also called Casmir-Polder forces in this type of context). A role for van der Waals forces was first explored by De Ninno in 2017 [80]. In his calculation, he assumes that water contains coherent domains with higher dielectric constant and lower density (akin to the “low density water phase” in the famous two-phase model of water) and “non-coherent domains” with much lower dielectric constant (ε≈13). The existence of such domains has not been experimentally validated for room temperature water, and molecular dynamics simulations have not discovered such coherent structures (see Reference [29] and references therein). A detailed analysis of the “quantum coherence domains” proposal was undertaken by Bier and Pravica, who concluded that Brownian motion forces wavefunctions to collapse and that such domains cannot exist in water [46]. Likewise, they showed that hydration shells around ions are limited to a nanometer in diamter [46].

The possibility that two objects of different composition may feel a repulsive force when submerged in a liquid was first realized by Hamaker in 1937 [81]. The full theory for such forces, for arbitrary dielectric media, was worked out by Lifshitz in 1954 [82]. Lifshitz’s equations allow for a repulsive force between two objects if the dielectric susceptibility of the medium between the two plates is intermediary between the two. Calculations using Lifshitz theory show that the finite size of the slabs does not effect the repulsion between them [83,84]. Having free electrons, the dielectric constant of metals is extremely high (for instance Milling take the dielectric constant of gold to be 300) [85]. The dielectric constant of water is 78 and the dielectric constant of a polystyrene microsphere is about 2.5 (other plastic microspheres should have dielectric constants between 1.5 and 3). Thus, the metal-microsphere-water system obeys the conditions necessary for Casmir Pollard repulsion.

Most studies of the repulsive van der Waals force have used liquids other than water, likely due to the fact that water is easily contaminated with charge bearing solutes which can confound such experiments. The effect is also larger in nonpolar liquids than polar ones [85]. Munday et al. (2009) have reported a repulsive Casmir force between a gold plate and a silica sphere submerged in bromobenzene [86]. Similar repulsion has been found in follow up work with cyclohexane and other liquids [87,88]. Milling et al. (1996) measured the force between a gold sphere and PTFE block submerged in several liquids, including water [85]. While their results for water were neutral/inconsistent (both weakly attractive and weakly repulsive forces were observed), their theoretical calculation indicates that the vdW force in water should be repulsive [85]. There is clearly room for improvement in here, as the sign of the Hamaker constant predicted by theory only matches the sign found by experiment in 3/10 cases, suggesting an issue either with the theoretical calculation or experiments. Milling et al. note that the discrepancies most likely arise on the theoretical end as due to incomplete knowledge about the high frequency (UV) dielectric function of the materials involved, as the entire dielectric spectrum is required for the calculation [85].

One issue with this theory though is that retardation effects can diminish the van der Waals force starting at just a few nanometers of separation [89,90]. Retardation effects become important when the travel time due to the speed of light becomes similar the timescale (period) of polarization fluctuations which underlie the van der Waals force. Under retardation the force changes from falling as $1/r^{7}$ to $1/r^{8}$. However, Isrealachvili notes that here is also a non-retarded zero frequency component to the vdW force which persists to large separations [91]. According to Isrealachvili, the actual progression of the vdW force may be from $1/r^{7}\to 1/r^{8}\to 1/r^{7}$ [91].

The growth of the EZ zone with laser light [38] or MHz frequency electromagnetic fields [92] may be due to an induced van der Waals repulsion, although there may be a more prosaic explanation. It has been shown that the van der Waals forces between silver nanoparticles can be enhanced by radiation, since electromagnetic radiation induces fluctuating dipole moments in the particles. The possibility for light-driven enhancement of repulsive van der Waals forces has been shown theoretically by Rodríguez-Fortuño et al. [93]. While these considerations are for metal nanoparticles, the polarizability of plastic (especially functionalized plastic) means such induced dipoles moments may be possible. Further theoretical study is needed to clarify this matter.

### 5.3. Other Possible Mechanisms and Experimental Confounds

Huszár et al. have investigated two other possible explanations for EZ-formation [3].

- Dissolution of Nafion, during which polymer strands diffusing out of the gel push the beads away from the surface.
- A “brush mechanism” in which closely spaced long elastic polymer strands keep the beads away by entropic forces.

Close inspection of gel showed that it does not loose mass, and an atomic force microscopy (AFM) study of the surface shows that there are no long strands hanging out, so they ruled out both of these mechanisms.

Bunkin et al. analyzed the swelling of Nafion with photo-luminescent UV spectroscopy, which detects the terminal sulfonic groups on Nafion fibers [94]. Notably, they observed there are fibers which extend out into the liquid; for deuterium this matched roughly the exclusion zone distance observed in Pollack’s experiments. The degree to which these fibers penetrate the liquid appears to be very sensitive to the presence of deuterium. As noted before, water-swollen Nafion absorbs UV strongly at 270 nm [95,96].

Apart from these two effects, there are other possible effects that can contaminate microsphere systems and confound experiments. Plastic nanospheres can be easily contaminated with charge bearing groups. In the case of polytetrafluoroethylene (PTFE) these may include “residual carboxylic groups from the polymerization process” [85]. Referring to research that uses plastic microspheres Horinek et al. note “these systems are notoriously plagued by secondary effects, such as bubble adsorption and cavitation effects or compositional rearrangements” [97]. As an example, the discovery of an ultra-low frequency Debye relaxation in water, for instance, was later show to be due to microbubble contamination [98]. There is also growing research showing that the removal of nanobubbles from water can be very challenging. This is especially true when they are adsorbed on surfaces. As noted before, the introduction of degassing methods reduced the appearance of the peak at 270 nm which Pollack attributes to EZ water [47]. Thus careful degassing should be a key part of any research on EZ water going forward.

Finally, in passing we note that Chaplin has a theory which he calls “self-generation of colligative properties” [99]. The basic idea is an “osmotic effect” can be generated near hydrophilic surfaces, since the water molecules very close to the surface are moving slower, effectively resulting in lower temperature water near the surface. Chaplin predicts that an even larger osmotic effect should occur near nanobubble’s surfaces, due to “surface teathered” solutes near or at the nanobubble air-water interface [100]. Chaplin’s theory will require carefully designed experiments to test.

## 6. Conclusions

In this review we noted several major problems with the theory that water in the EZ undergoes a phase change or significant reordering. We presented new results from neutron beam radiography which do not support the idea of a higher density phase and discussed how flaws were discovered in Pollack’s birefringence measurements, which have been used as evidence for a structure change. Schurr’s theory of macroscopic chemotaxis presents a compelling alternative theory which can explain experimental findings which Pollack’s theory cannot, such as the precise time course of EZ growth, pH gradients emanating from the surface of Nafion, and the decaying forcefield measured by experiments with optical tweezers [1,3,6,11,70]. There are still many open questions about exclusion zones. The findings of EZs near different metal surfaces need to be better replicated and elaborated, as some attempts to replicate these findings have failed [9]. Many findings from Pollack’s lab still need to be replicated by independent groups, in particular the growth of the EZ with laser irritation and the exclusion of salt. Both of these phenomena, if genuine, are in need of further explanation. Likewise, Rohani & Pollack have observed anomalous flow in Nafion tubes, and understanding this phenomena may shed light on the ion dynamics around Nafion or here-to undiscovered experimental confounds [101]. A more complete understanding of the mechanisms behind EZ phenomena will assist in understanding their possible roles in biology as well as their possible engineering applications such as microfluidics and filtration.

## Figures and Tables

**Table 1:** Some of the reported properties of the exclusion zone water.

Measured Property	EZ Water Value	Bulk Value	References
refractive index	1.46	1.33	Bunkin et al., 2013 [7]
T${}_{2}$ relaxation time	27.2 ± 0.4 ms	25.4 ± 1 ms	Zhen et al, 2006 [12]
electric potential near surface	−120 to −200 mV	0 mV	[12,33,39]
